# Telemedicine for prehospital respiratory emergencies in a retrospective quality analysis

**DOI:** 10.1038/s41598-025-01850-1

**Published:** 2025-05-22

**Authors:** Alexander Beierle, Syrina Beierle, Mark Pitsch, Despina Panagiotidis, Jan Larmann, Stefan K. Beckers, Marc Felzen, Hanna Schröder

**Affiliations:** 1 Department of Anesthesiology, Medical Faculty RWTH Aachen University, University Hospital RWTH Aachen, Pauwelsstrasse 30, 52074 Aachen, Germany; 2 Aachen Institute for Rescue Management & Public Safety, City of Aachen and University Hospital RWTH Aachen, Pauwelsstrasse 30, 52074 Aachen, Germany

**Keywords:** Medical research, Preclinical research

## Abstract

Respiratory distress is a common reason for emergency medical service (EMS) physicians to be prehospitally involved. While the availability of telemedical EMS systems increases continuously, there is a gap in research regarding respiratory emergencies in the context of prehospital telemedicine. The aim of this study is to evaluate the quality of care provided in prehospital respiratory emergencies, managed through a tele-EMS system with a specialized EMS physician. Tele-EMS physician missions from 01/01/2019 to 12/31/2021 in Aachen, Germany, were analyzed. Adult patients presenting with dyspnea, peripheral oxygen saturation < 94%, respiratory rate > 19/min, or any combination of these factors, were included (n = 2234). Data were derived from mission protocols recorded by the attending tele-EMS physicians. Significant changes (*p* < 0.001) in vital parameters towards physiological ranges were observed. For the most common diagnoses, a significant improvement in patient condition was achieved with the use of appropriate medications. In 14 cases (0.63%), an onsite-EMS physician was requested. These cases were confirmed to involve unstable patients who required intervention of an onsite-EMS physician. The tele-EMS physician effectively bridged the time until arrival of the physician. Overall, the study demonstrated that respiratory emergencies were effectively managed using the tele-EMS physician system.

## Introduction

Conditions leading to severe respiratory distress are a common cause of death in adults and serve as a frequent trigger for emergency response^1^.

Firstly, reasons for this include demographic changes with an aging population and the associated increasing morbidity. In contrast, there is a shortage of qualified staff in emergency medical services (EMS), rising response times in rural areas leading to delayed patient care, and increasing economic pressure. Secondly, this patient group poses a challenge for prehospital emergency teams, as there are numerous differential diagnoses to be considered, leading to diverse—sometimes even conflicting—therapeutic options^2,3^. Studies have shown a correlation between misdiagnoses and increased mortality in emergency patients with respiratory problems^4,5^.

All these factors may impact the outcome of the patients to be treated^6–8^. To counteract these problems and provide a possible solution, telemedicine is increasingly being integrated into prehospital emergency care^9^. During the COVID-19 pandemic, it was demonstrated that various technological systems were used in the out-of-hospital care of patients. For example, the benefit of ultrasound performed by nurses on patients at home, with the support of a telemedicine-accessible physician, was shown^10^.

The increasing availability of telemedical support for EMS has not yet been researched in the specific context of respiratory emergencies. To address the disparity between a growing number of aging patients with respiratory problems and the shortage of skilled personnel, along with an increased number of emergency calls, it is imperative to investigate prehospital emergency telemedicine in relation to this specific patient group^11^. There are currently only a few studies that consider telemedicine as one of several pillars of the treatment of patients with respiratory emergencies. For example, one study demonstrated that telemedical respiratory examinations are feasible and reliable in young children^12^. Additionally, another study indicates that the use of telemedicine in the management of patients with asthma led to a reduction in exacerbations^13^. However, there is a lack of data that examines the quality of care for this adult patient group as comprehensively as possible using telemedicine in the prehospital setting.

The main objective of this study focuses on assessing the quality of care provided by a telemedicine emergency medical service (tele-EMS) physician system for patients with respiratory emergencies. The aim is to determine whether the affected patients received adequate treatment and experienced a positive outcome under the administered therapy, until handoff to the hospital. Along with this, the various diagnoses that lead to respiratory distress are being examined in detail, and the respective management by the tele-EMS physician is being analyzed.

## Materials and methods

### Study design

This retrospective study examines tele-EMS physician missions in Aachen, Germany, from the years 2019, 2020, and 2021. The data were made available on 10/30/2022.

### Structure and setting of telemedical system in the city of Aachen, Germany

According to the statistical Yearbook 2020–2021 of Aachen, in 2019, 258,816 people lived in Aachen, which, with an area of 160.8 square kilometers, resulted in an average population density of 1609 inhabitants per square kilometer. The population decreased only marginally during the study period, by 228 people, to a total of 258,588 inhabitants. In these years, 10,761 tele-EMS interventions were reported. This corresponds to an incidence of 4158 per 100,000 inhabitants.

The German EMS is characterized by the routine involvement of emergency physicians in prehospital care. The tele-EMS physician system in Aachen is based on two pillars: the tele-EMS physician center and the specially equipped ambulances. On one hand, the tele-EMS physician is located in the tele-EMS physician center. This center is staffed 24/7, and the tele-EMS physician on duty is responsible for immediate remote response to consultation requests from the on-scene teams. The workstation is specially equipped and includes multiple monitors, and data linkage providing the tele-EMS physician with access to live-transmitted vital signs, visual material from the scene, the location of the on-site team, documentation software, and full access to all local treatment protocols^14–16^. On the other hand, the ambulances are equipped with a communication unit that ensures the transmission of the aforementioned data to the tele-EMS physician center. Additionally, a smartphone can be used for photo transmission, and a high-resolution camera in the ambulance can be remotely accessed for video transmission^14,17^.

When considering the personnel involved, two professional groups are regarded as essential components of tele-EMS care. First, the tele-EMS physician: In Germany, the requirements for obtaining tele-EMS physician qualification are regulated. It is required that the physician has completed at least 500 missions as an on-scene EMS physician, has completed a medical specialization in a field relevant to emergency medicine, and holds a course certificate in intensive care transport. Local regulations may require additional qualifications^18^. Second, paramedics with a three-year vocational training: Paramedics working with tele-EMS undergo an additional one-day training course on the use and application of the specific tele-EMS system in their EMS district. Paramedics autonomously decide whether to consult a tele-EMS physician or to independently manage the emergency according to local protocols^14,19^.

### Inclusion and exclusion criteria

**Fig. 1:**
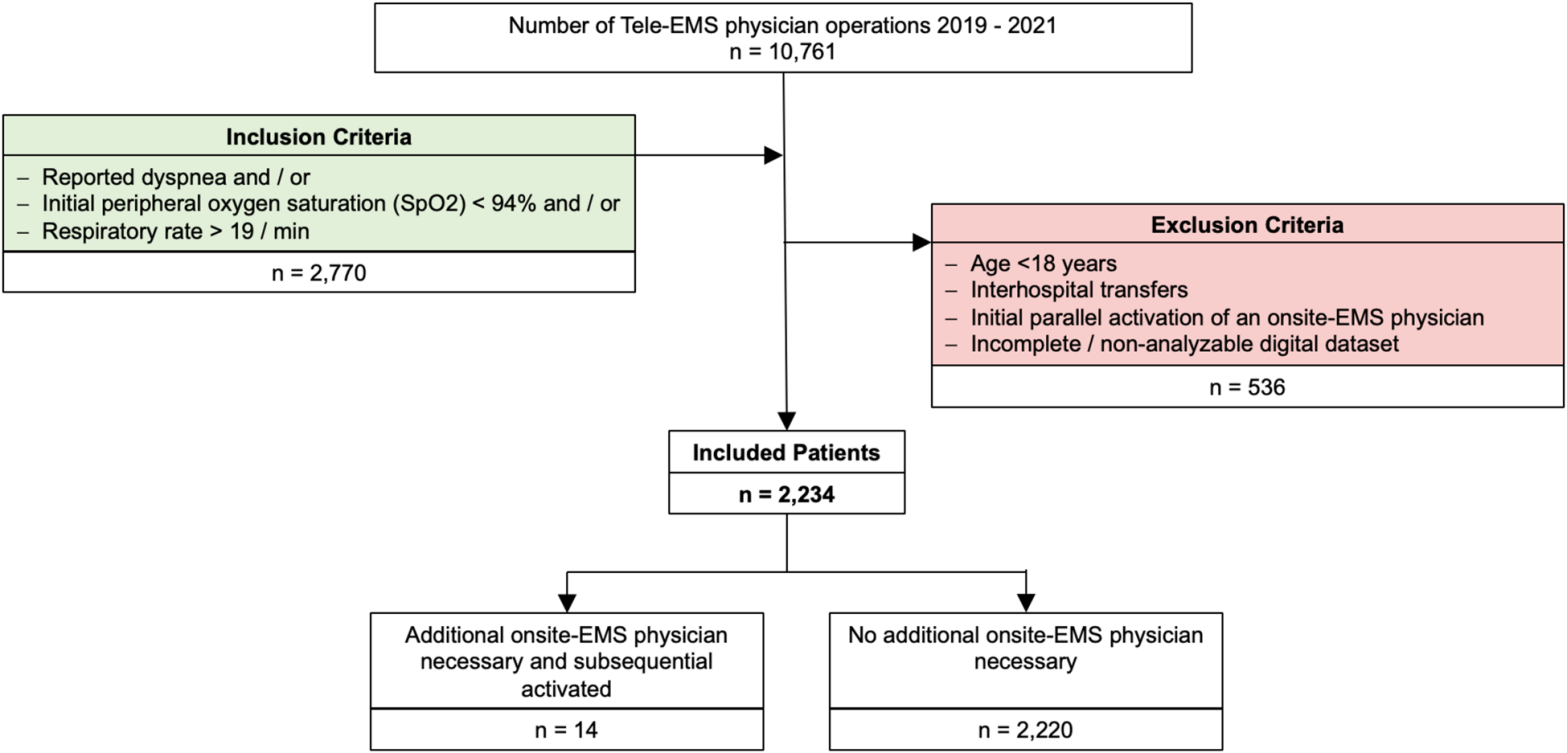
Inclusion and exclusion criteria. EMS = emergency medical service; SpO_2_ = peripheral oxygen saturation.

The data consists of digital tele-EMS physician protocols. Before including the data in the statistical analysis, plausibility checks were carried out. During this period, the tele-EMS physician was consulted on 10,761 missions. The inclusion and exclusion criteria are shown in Fig. 1.

To include all patients respiratory emergencies were defined as follows: patients who experienced dyspnea, had a peripheral oxygen saturation (SpO_2_) below 94%, had a respiratory rate above 19 / min, or any combination of these factors. A saturation below 94% was chosen because it corresponds to current standards, literature, and guidelines^20^. The same applies to the inclusion of patients with a respiratory rate greater than 19 / min to ensure that no patients with respiratory emergencies are overlooked. Subsequently, patients were excluded if they were under 18 years of age, underwent inter-hospital transfers, or were involved in incidents where an on-site EMS physician was simultaneously dispatched. This approach resulted in a study group of 2234 patients.

### Examined factors and outcome variables

The following demographic data were included: age, gender, National Advisory Committee for Aeronautics (NACA) score, and diagnosis. The NACA score is a system used to evaluate medical emergencies and is applied in many European countries, ranging from NACA 1 (minor disturbance) to NACA 7 (death) (NACA 2—moderate disturbance, NACA 3—severe but not life-threatening disorder, NACA 4—potentially life-threatening, NACA 5—acute risk of death, NACA 6—cardiac arrest)^14^.

To assess whether the tele-EMS physician system was able to effectively manage the patients’ respiratory issues, the primary outcome variables considered were respiratory rate in 1/min, SpO_2_ in %, heart rate in 1/min, and systolic blood pressure in mmHg. The initial measured vital signs were compared with the last measurements. In addition, oxygen administration was examined in greater detail, and the correlation between SpO_2_ < 94% and oxygen administration was analyzed.

In order to evaluate which conditions related to respiratory emergencies could be managed effectively by the tele-EMS physician, the diagnoses were categorized into primary cardiovascular, primary respiratory, primary neurological / psychological, primary other focus, and trauma. These diagnostic groups reflect that dyspnea is a common leading symptom of various medical conditions. Diagnoses were assigned accordingly. Diagnoses that could not be clearly classified into one of these groups, were assigned to the primarily respiratory group, as this was the main focus of this study (e.g., anaphylactic reactions with cardiovascular and respiratory manifestations). This resulted in the following clusters: For primarily cardiovascular group, the diagnoses included acute coronary syndrome (ACS), hypertensive crisis, arrhythmia, (pre-)syncope, decompensated heart failure, thoracic pain, pulmonary embolism, and anemia. For primarily respiratory group, the diagnoses encompassed respiratory infections and pneumonia, obstructive lung diseases with exacerbation, unspecified dyspnea, allergic reactions, pulmonary edema, aspiration, and pneumothorax. Lastly, for other causes, the diagnoses involved pain syndromes, gastritis, general deterioration of condition, dehydration, intoxications, urinary tract infections, hypo- or hyperglycemia, and palliative care. Additionally, the initial and most recently measured vital parameters (Respiratory rate, SpO_2_, heart rate, systolic blood pressure) of the individual diagnostic groups are compared.

To enable a more detailed investigation of as many treatments as possible, the two most common diagnoses within the cardiovascular group and the two most common diagnoses within the respiratory group were further analyzed. This approach ensures a detailed examination of the majority of treated patients without compromising clarity. Thus, the following diagnoses were reviewed within the primary cardiovascular group: ACS and hypertensive crisis. For ACS, the administration of heparin, aspirin, and oxygen was examined in greater detail. For patients with hypertensive crises, the administration of urapidil and oxygen was examined, as this is part of the local protocol.

Under the primary respiratory group, respiratory infections / pneumonia and obstructive lung diseases were analyzed. For respiratory infections, the focus was placed on oxygen administration. Due to the variability of infections (e.g. COVID-19, pneumonia) and the corresponding spectrum of treatments, this analysis focused on the evaluation of oxygen administration, as no anti-infective medication is carried in the emergency medical services of the Aachen region. In cases of obstructive lung diseases, the administration of ipratropium bromide, salbutamol, prednisone, and oxygen were evaluated.

Lastly, we analyzed a small number of cases in which an onsite-EMS physician was secondarily dispatched (n = 14). In these cases, the onsite-EMS physician provided a handwritten intervention protocol, which served as data source. In 4 missions, the onsite-EMS physician was affiliated outside the city of Aachen and these protocols were unavailable for analysis. Consequently, 10 protocols are used as the basis for this subgroup analysis. Demographics, NACA score, diagnostic group, and vital parameters were analyzed.

### Statistical analysis

The digital records underwent plausibility checks to reduce and correct obvious errors before conducting statistical analyses. These checks were performed by the authors. In the individual analyses, patients with missing and irreproducible relevant data were excluded. The statistical analyses were performed using Excel (Microsoft, Washington, USA, 2023, Microsoft 365, version 16.75.2) and Stata (Stata Corp LLC, Texas, USA, 2016, version 13.1). Depending on the specific research question, the exact test according to Fisher, Pearson’s chi-squared test, and paired and unpaired t-tests were applied. Two-sided *p*-values < 0.01 were considered significant and the 95% confidence intervals (CI) were reported.

### Ethics approval and consent to participate

The Ethics Committee at the RWTH Aachen University, Faculty of Medicine (Pauwelsstraße 30, 52074 Aachen, Germany) reviewed the analyses and identified no constraints (Approval number: 180/18). The requirement for obtaining informed consent was waived by both the Ethics Committee at the RWTH Aachen Faculty of Medicine (Pauwelsstraße 30, 52074 Aachen, Germany; Head: Prof. Hausmann), the Center for Translational and Clinical Research (CTC-A) of RWTH Aachen University and the relevant data protection officers, as this retrospective analysis was conducted anonymously as part of the legally mandated quality assurance duties of municipal authorities. All methods in this study were performed in accordance with the relevant guidelines and regulations. Access to the data for this study was granted on 10/30/2022, and data processing was carried out using a pseudonymized dataset.

## Results

### Study population

**Fig. 2:**
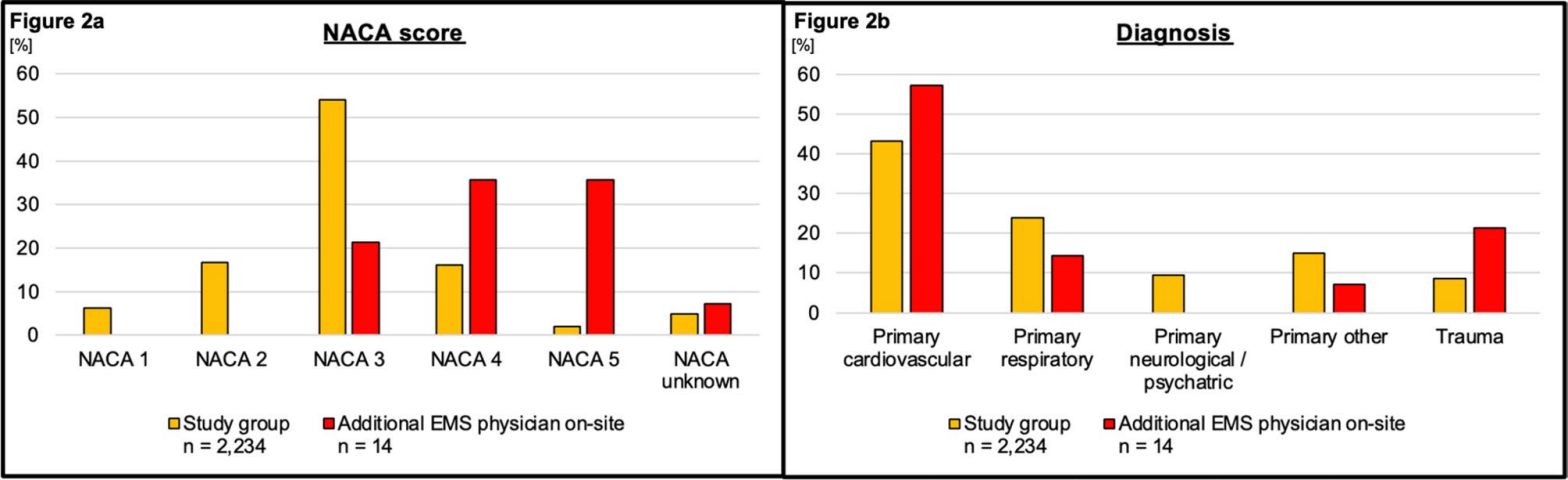
(**a**) NACA score. (**b**) Diagnosis. EMS = emergency medical service; NACA = National Advisory Committee for Aeronautics.

During the 3-year study period (1/1/2019–12/31/2021), the tele-EMS system was involved in 10,761 missions. A total of 2234 patients were included in this study. Therefore, 20.76% (2234/10,761) of all patients requiring a tele-EMS physician were examined. 1208 patients were included due to reported dyspnea, 899 were included due to documented SpO_2_ < 94%, 540 due to documented respiratory rate > 19/min. This results in 2234 included cases after accounting for duplicates, where patients met multiple criteria. The patients examined had a mean age of 67 years (standard deviation (SD) 19.41), and 46.73% (1044/2234) were female. Table 1 presents the demographics of all included patients (study group, n = 2234) along with the demographics of patient clusters (cardiovascular, respiratory, neurological / psychiatric, other primary conditions, and trauma). Figure 2 illustrates the NACA score and the diagnostic groups for the entire study cohort (yellow), as well as the NACA score and diagnostic cluster specifically for patients for whom an EMS physician was subsequently requested on-site (red).

**Table 1:** Patient demographics and allocation due to categorized leading diagnosis groups.

	Study Group (n = 2234)	Primary cardiovascular (n = 965)	Primary respiratory (n = 533)	Primary neurological / psychiatric (n = 211)	Primary other (n = 334)	Trauma (n = 191)
Age, mean (standard deviation (SD))	67 (19.41)	70 (16.68)	66 (19.54)	64 (22.66)	64 (20.97)	67 (22.89)
Sex female, n (%)	1044 (46.73)	454 (47.05)	225 (42.21)	108 (51.18)	165 (49.41)	92 (48.17)
Sex male, n (%)	1009 (45.17)	436 (45.18)	260 (48.78)	84 (39.81)	143 (42.81)	86 (45.03)
Sex unknown, n (%)	181 (8.10)	75 (7.77)	48 (9.01)	19 (9.01)	26 (7.78)	13 (6.81)
Number 2019, n (%)	584 (26.14)	302 (31.30)	106 (19.89)	47 (22.27)	85 (25.45)	44 (23.04)
Number 2020, n (%)	796 (35.63)	309 (32.02)	231 (43.34)	72 (34.12)	110 (32.93)	74 (38.74)
Number 2021, n (%)	854 (38.23)	354 (36.68)	196 (36.77)	92 (43.61)	139 (41.62)	73 (38.22)

### Vital parameters

**Fig. 3:**
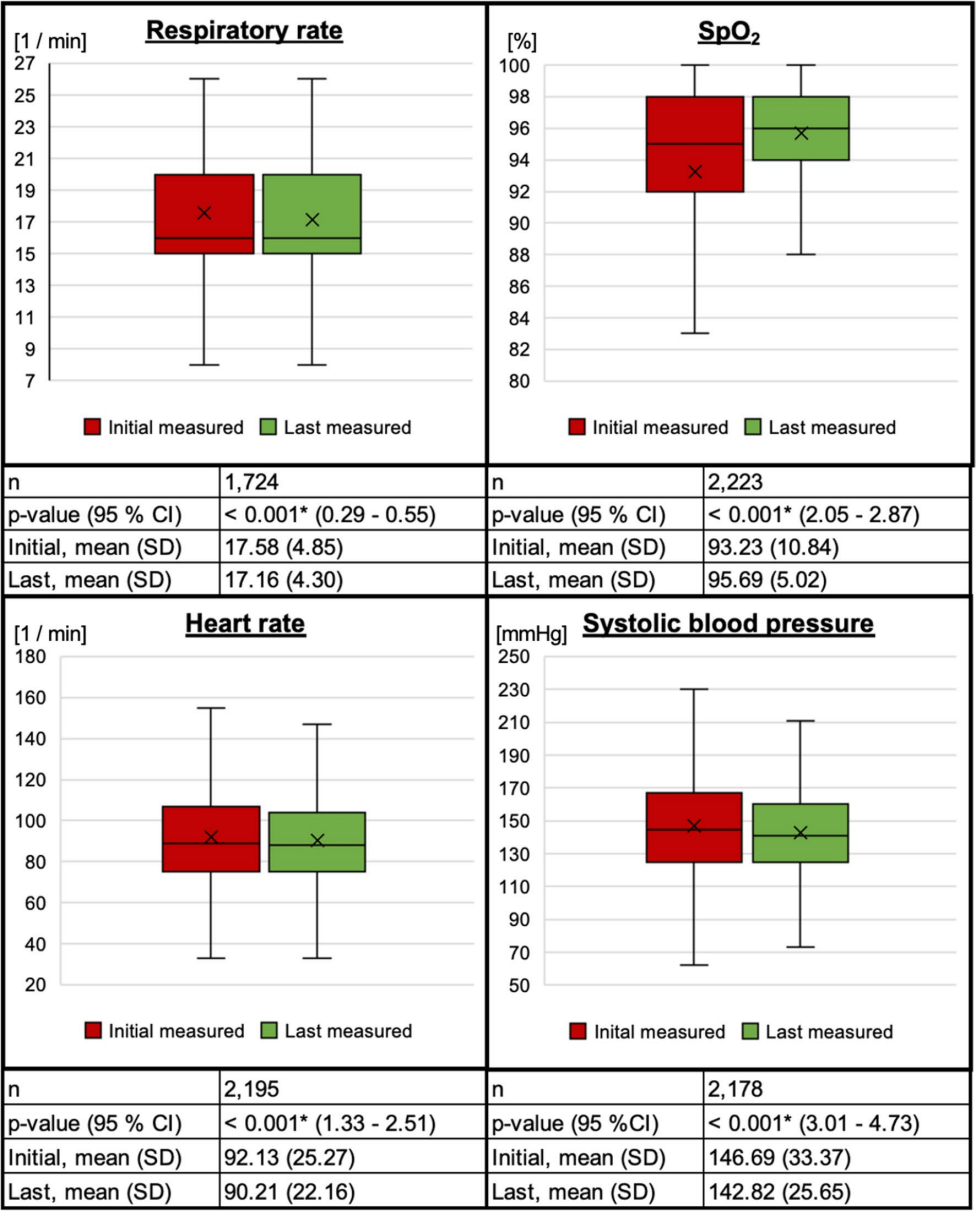
Vital signs. CI = confidence interval; SpO_2_ = peripheral oxygen saturation; * represents significant difference (*p* < 0.001).

When evaluating the vital parameters, we compared the initially measured values with the last measured values. The difference between the initially measured respiratory rate and the last measured was significant (*p* < 0.001; 95% CI 0.29–0.55, n = 1724). A significant difference in oxygen saturation was observed between the initial and the last measurements (*p* < 0.001, 95% CI 2.05–2.87, n = 2223). Regarding heart rate, significant differences were also identified: Initial and last (*p* < 0.001, 95% CI 1.33–2.51, n = 2195); The measured systolic blood pressure differed significantly between the initial and last measured values (*p* < 0.001, 95% CI 3.01–4.73, n = 2178). Overall, 22.74% (508/2234) of the patients received oxygen therapy, either via nasal cannula or oxygen mask. The administration of oxygen was significantly associated with SpO_2_ < 94% (*p* < 0.001, n = 2234). Figure 3 illustrates the detailed variations in vital signs.

When examining the respiratory rate, it is notable that the boxplots appear identical, even though the paired t-test indicates a significant difference. This is attributable, in part, to the large sample size and the small absolute difference observed. Thus, this indicates statistical significance despite a clinically insignificant difference in respiratory rate.

### Diagnostic groups

**Fig. 4:**
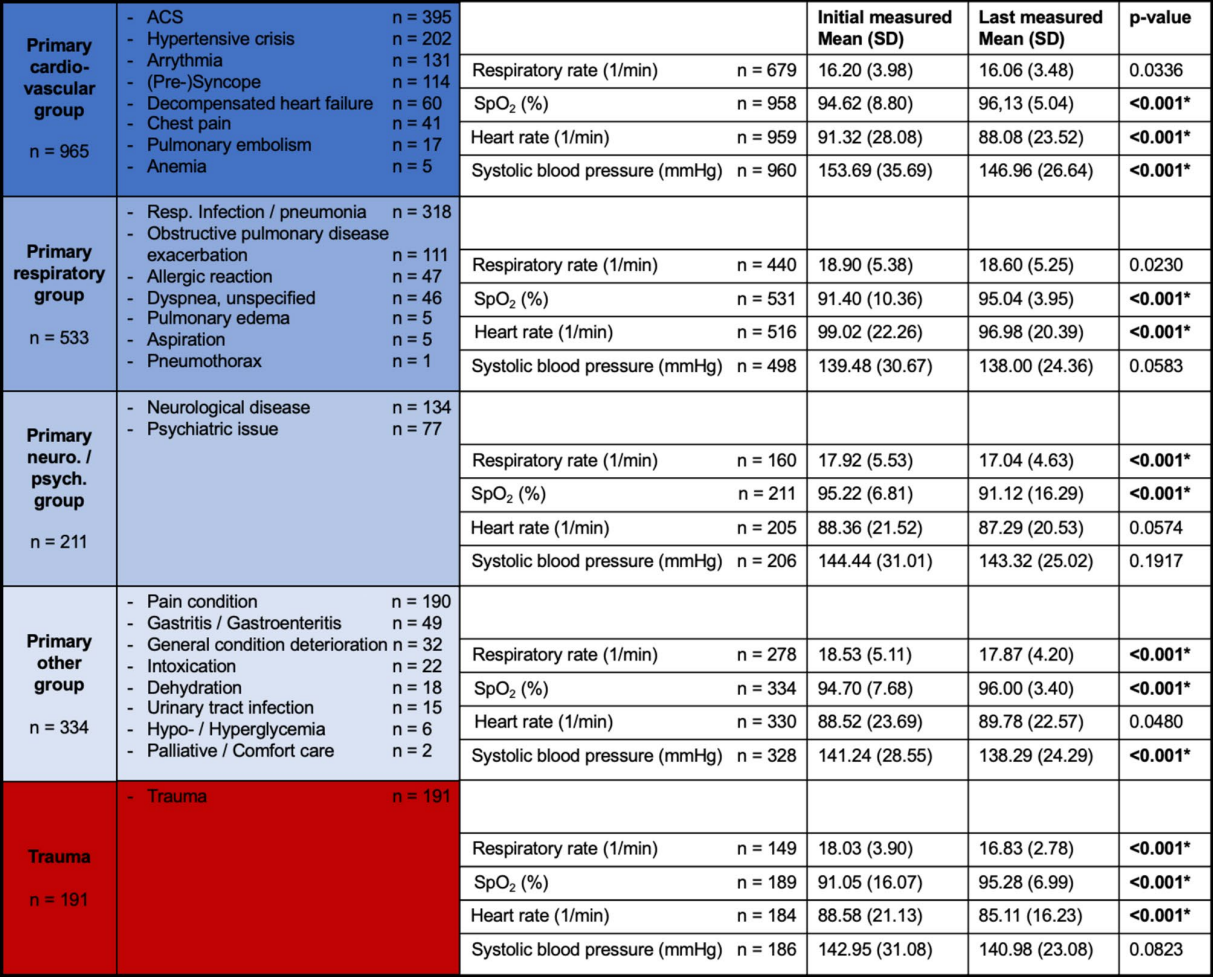
Categorization of diagnoses and according measurements. ACS = acute coronary syndrome; SpO_2_ = peripheral oxygen saturation; * represents significant difference (*p* < 0.001).

For the assessment of each diagnostic group, both the initial and final recorded vital parameters were compared. Figure 4 summarizes the individual diagnostic groups, the measured vital parameters, and their comparisons. Table 2 illustrates the computed differences between the initial and last measured vital parameters, compared across the individual diagnostic groups.

**Table 2:** Comparison of differences in vital parameters across diagnostic groups.

p-value (CI)	Resp.	Neuro.	Other	Trauma	Resp.	Neuro.	Other	Trauma
Initial measured respiratory rate					Last measured respiratory rate			
Cardio. n = 697	< 0.001** (2.16–3.25)	< 0.001** (0.98–2.47)	< 0.001** (1.73–2.93)	< 0.001** (1.13–2.53)	< 0.001** (2.03–3.05)	< 0.01* (0.34–1.62)	< 0.001** (1.29–2.32)	< 0.01* (0,17–1.36)
Resp. n = 440		0.0249 (0.00–1.97)	0.1756 (-0.42–1.17)	0.0338 (-0.06–1.81)		< 0.001** (0.63–2.48)	0.0252 (0.00–1.46)	< 0.001** (0.88–2.65)
Neuro. n = 160			0.1234 (-0.42–1.63)	0.5781 (-1.19–0.97)			0.0284 (-0.02–1.68)	0.3149 (-0.65–1.07)
Other n = 278				0.1499 (-0.45–1.44)				< 0.01* (0.29–1.79)
Initial measured SpO_2_					Last measured SpO_2_			
Cardio. n = 958	< 0.001** (2.23–4.22)	< 0.001** (1.94–5.09)	0.4433 (-0.99–1.14)	< 0.001** (1.96–5.19)	< 0.001** (0.59–1.59)	0.0133 (0.11–1.72)	0.3340 (-0.46–0.71)	0.0244 (0.00–1.70)
Resp. n = 531		0.3870 (-1.68–2.26)	< 0.001** (2.02–4.60)	0.3667 (-1.67–2.37)		0.3301 (-0.61–0.96)	< 0.001** (0.45–1.48)	0.2837 (-5.8–1.06)
Neuro. n = 211			< 0.001** (1.56–5.63)	0.4849 (-3.13–3.25)			0.0375 (-0.08–1.65)	0.4640 (-1.30–1.42)
Other n = 334				< 0.001** (1.61–5.70)				0.0566 (-0.17–1.62)
Initial measured heart rate					Last measured heart rate			
Cardio. n = 959	< 0.001** (4.89–10.50)	0.0772 (-1.12–7.05)	0.0519 (-0.57–6.18)	0.1041 (-1.53–7.02)	< 0.001* (6.50–11.31)	0.3282 (-2.69–4.26)	0.1261 (-1.21–4.61)	0.0512 (-0.59–6.52)
Resp. n = 516		< 0.001** (7.08–14.23)	< 0.001** (7.34–13.65)	< 0.001** (6.74–14.14)		< 0.001** (6.38–13.00)	< 0.001** (4.26–10.14)	< 0.001** (8.60–15.13)
Neuro. n = 205			0.4686 (-3.83–4.16)	0.4596 (-4.04–4.48)			0.0999 (-1.32–6.30)	0.1256 (-1.54–5.89)
Other n = 331				0.4887 (-4.06–4.18)				< 0.01* (0.95–8.37)
Initial measured systolic blood pressure					Last measured systolic blood pressure			
Cardio. n = 960	< 0.001** (10.52–17.90)	< 0.001** (4.00–14.51)	< 0.001** (8.18–16.72)	< 0.001** (5.25–16.24)	< 0.001** (6.15–11.76)	0.0361 (-0.33–7.61)	< 0.001** (5.38–11.95)	< 0.01* (1.87–10.08)
Resp. n = 498		0.0261 (-0.05–9.96)	0.2028 (-2.40–5.93)	0.0951 (-1.73–8.66)		< 0.01* (1.32–9.31)	0.4335 (-3.14–3.72)	0.0744 (-1.07–7.04)
Neuro. n = 206			0.1122 (-1.96–8.35)	0.3176 (-4.68–7.66)			0.0120 (0.66–9.38)	0.1699 (-2.46–7.13)
Other n = 327				0.2649 (-3.62–7.02)				0.1138 (-1.69–7.07)

Cardio. = Primary cardiovascular group;

Neuro. = Primary neurological / psychiatric group;

Other = Primary other group;

Resp. = Primary respiratory group;

SpO_2_ = peripheral oxygen saturation;

*Represents significant difference (*p* < 0.01);

**Represents significant difference (*p* < 0.001);

Significant values are in bold.

Upon examination of oxygen administration, the following values were identified: 153 patients with primary cardiovascular conditions, 312 with primary respiratory conditions, 33 with primary neurological / psychiatric conditions, 135 with primary other conditions, and 40 trauma patients received oxygen. Across all diagnostic groups, a significant association was found between oxygen administration and SpO_2_ < 94% (*p* < 0.001).

Regarding primarily cardiovascular group, the two most frequent diagnoses were examined in more detail. In cases of ACS, a significant correlation was observed between this diagnosis and the administration of aspirin and / or heparin (*p* < 0.001, n = 2234). Additionally, 56 patients received oxygen, which was significantly associated with oxygen saturation below 94% (*p* < 0.001, n = 395). In patients with hypertensive crises, the mean systolic blood pressure was reduced from 191.56 (SD 28.52) to 171.56 (SD 21.76). 138 of these patients received urapidil showing a significant association between the diagnosis hypertensive crisis and receiving urapidil (*p* < 0.001, n = 2234). 8 patients with peripheral oxygen saturation below 94% were also given oxygen (*p* < 0.001, n = 202).

For primary respiratory group, two diagnoses were analyzed separately: infections / pneumonia and obstructive lung diseases. For patients with pneumonia/infections, the oxygen saturation increased on average from an initial value of 91.16% (SD 9.73) to 94.52% (SD 3.85) at the final measurement. 117 patients received oxygen therapy, 99 of whom showed SpO_2_ < 94%. This represents a significant association between oxygen administration and SpO_2_ < 94% in this subgroup (*p* < 0.001, n = 318). In cases of obstructive lung diseases, a significant association was found between oxygen administration and SpO_2_ < 94% (*p* < 0.001, n = 111). 100 patients received oxygen therapy. In 88 cases, ipratropium bromide and / or salbutamol and / or prednisone were administered. All these interventions were found to be significantly associated with this diagnosis (*p* < 0.001, n = 2234).

### Missions with additional emergency physician deployment

Regarding the analysis of tele-EMS consultations where a physical EMS physician was requested, the following data were collected. In 0.63% (14/2234) of the cases, an additional EMS physician was requested on-site. The patients had an average age of 67 years (SD 14.93), and 57.14% (8/14) of them were female. Five consultations occurred in 2019, 6 in 2020, and one in 2021. The NACA score and the diagnostic groups are summarized in Fig. 2. The initial vital parameters presented with the following mean values: respiratory rate 17.27/min (SD 4.78), SpO_2_ 92.33% (SD 4.29), heart rate 99.42/min (SD 22.42), and systolic blood pressure 140.83 mmHg (SD 44.76). During the final measurement, the following values were recorded: respiratory rate 17.72/min (SD 5.27), SpO_2_ 94.33% (SD 4.79), heart rate 96.42/min (SD 23.52), and systolic blood pressure 143.75 mmHg (SD 46.77). In all cases, the patients were classified as hemodynamically or respiratory unstable. In two cases, the patients underwent prehospital endotracheal intubation. In another two cases, the patients presented with ST-elevation myocardial infarction. In other instances, the EMS physician was called to the scene due to difficulties in establishing intravenous access, escalating already initiated analgesia, and initiating non-invasive ventilation.

## Discussion

We analyzed data from patients who presented with respiratory emergencies and were treated by the tele-EMS physician system. The data revealed a wide range of diagnoses that involved the signs and symptoms of dyspnea, peripheral oxygen saturation levels below 94%, respiratory rates above 19 / min, or any combination of these. Overall, 20.76% (2234/10,761) of all tele-EMS physician system cases during the study period were included. This indicates that the investigated patient group represented a significant proportion of those treated by the system. This finding aligns with the current literature, which emphasizes that patients with respiratory emergencies constitute a substantial portion of the prehospital EMS care population^21,22^.

An important question is whether this diverse patient group was adequately managed using the tele-EMS physician system. It is well established that the highest standards of patient safety can be achieved through guideline-adherent practices and structured processes^23–26^. These standards, however, are predominantly based on the physical presence of a physician. Furthermore, in the prehospital setting, decisions are influenced by numerous factors, and the resources available in an in-hospital environment are not readily accessible^27^. Are these multifactorial decisions and processes sustainable when managed by a remotely located EMS physician?

It is important to note that the traditional paternalistic model, where "the doctor knows best," has largely been replaced by guideline-based medicine, which is grounded in evidence or at least expert consensus^24^. Guideline-based medicine is carried out by various professional groups within the medical field. In the context of this study, the focus is on the tele-EMS physician system. However, it is also emphasized that the on-site team is an integral part of the tele-EMS physician system. This team consists of paramedics and, in some cases, on-site EMS physicians. Previous studies have demonstrated that these professional groups, both in combination and independently, are capable of working in accordance with clinical guidelines, thereby contributing to patient outcomes^25,28,29^.

However, it cannot be determined whether, in the cases examined, the same guideline adherence would have been achieved by the on-site paramedic team alone. On-site paramedics utilize the tele-EMS physician system voluntarily. Consequently, consultation with the tele-EMS physician was typically initiated at the discretion of the paramedics, for instance, in instances of diagnostic ambiguity, when specific patient presentations necessitated physician advice, or when the patient’s clinical presentation did not conform to established standard operating procedures. The exclusion of respiratory emergencies managed exclusively by on-scene teams may introduce a potential selection bias. Future studies should address this question, as it would also facilitate the potential transferability of such systems to settings where no on-site EMS physician is present. For example, it could be investigated whether training paramedics in ultrasonography provides added value when combined with the tele-EMS physician system.

Furthermore, the requirements for working as a tele-EMS physician were outlined. Considering the declining number of on-scene deployments, the prerequisite of 500 missions may become increasingly difficult to achieve in the future. However, it should be considered that a considerable number of these 500 deployments do not necessitate technical or invasive measures^30^, suggesting that the declining number of on-scene deployments might not necessarily represent a relevant loss of experience. This could be one reason to reevaluate and potentially adjust the qualification criteria for tele-EMS physicians in order to ensure the long-term recruitment of experienced and qualified personnel.

### Vital parameters

The number of prehospital emergency cases involving dispatch categories related to respiratory emergencies remains consistently high in recent years^21,22^. These patients pose a significant challenge for attending EMS personnel. A wide range of diagnoses is associated with this clinical presentation, necessitating diverse treatment approaches^31,32^. In our study, 20.76% (2234/10,761) of all tele-EMS emergency cases were analyzed, representing a substantial proportion of total cases, consistent with current literature^31,33^. Furthermore, previous research has demonstrated that emergency patients with respiratory distress show high rates of in-hospital mortality^4^. This highlights the importance of thoroughly investigating this patient group and identifying potential deficits in their prehospital care, in line with the principles of an open safety culture and improved patient safety.

When evaluating vital parameters, significant changes were observed in peripheral oxygen saturation, systolic blood pressure, heart rate, and respiratory rate over the course of tele-EMS consultations. An analysis of these vital signs largely demonstrated a shift toward the respective desired target ranges. Studies conducted in in-hospital settings have demonstrated that vital signs provide valuable insights into the condition of patients. Moreover, when correctly interpreted, they can facilitate accurate and timely diagnosis and treatment^34^. Thus, maintaining vital signs within target ranges is associated with more stable patient conditions and improved outcomes. In the prehospital setting, it has been demonstrated that patients could be stratified, among other factors, based on their vital parameters, allowing differentiation between low, medium, and high-risk patients^35^. On average, the prehospital tele-EMS physicians together with on-scene paramedics were able to achieve target values in vital signs for their patients through the applied therapies, without a physician being physically present at the scene. This underscores the considerable potential of telemedicine, which has already been broadly demonstrated in other studies^16^. Its application in the management of respiratory emergencies thus represents a promising use case for this system. However, future studies should provide a comparative overview of the skills and resulting outcomes that on-site paramedics can achieve independently, and at which specific points the tele-EMS physician system contributes, as this cannot be fully determined from our data.

### Diagnostic groups

The two largest diagnostic groups identified are primary cardiovascular causes and primary respiratory causes. This distribution does not differ from other studies in the prehospital setting related to telemedicine, which also report cardiovascular cases as the predominant group^14,36^. Similarly, cardiovascular emergencies frequently dominate in prehospital emergency medicine without telemedicine systems^37^. Therefore, this study represents a realistic composition of common emergency scenarios and can serve as a robust basis for further investigations.

When comparing the individual diagnostic groups with each other, it becomes evident that both the respiratory rate and heart rate in the primary respiratory group differ significantly from most other groups (*p* < 0.001). This holds true for both the initial measurements and the last recorded values. Overall, respiratory and heart rates closer to the normal range were achieved in this diagnostic group. Similar observations apply to oxygen saturation in the primary cardiovascular diagnostic group, where significant differences are noted compared to the other groups. However, the results of the last recorded values also demonstrate a marked improvement.

When focusing on cases with a primary cardiovascular diagnostic group, the two dominant conditions identified are ACS and hypertensive emergencies. ACS is one of the most common conditions encountered in prehospital patient care^37^ and therefore, it is particularly important to examine guideline adherence and patient safety in this context. Studies have already demonstrated that prehospital operations utilizing tele-EMS systems exhibit a high level of adherence to guidelines, comparable to operations involving an on-site EMS physician^28^. The administration of aspirin and heparin was documented and correlated significantly with the diagnosis of ACS (*p* < 0.001). In addition, oxygen was administered when SpO_2_ < 94% (*p* < 0.001). These findings are consistent with the existing literature. Previous studies have shown guideline-compliant administration of aspirin, heparin, morphine, and oxygen using a tele-EMS physician system^8,28,38^.

The treatment of hypertensive emergencies is discussed second. In our study, we demonstrated that the systolic blood pressure was reduced. The final values were closer to the targeted range, while ensuring they did not fall below 20% of the initial measurement, following local and national protocols. Additionally, the administration of appropriate medications was shown to be significant (*p* < 0.001). One study has shown that for hypertensive crises and emergencies in tele-EMS deployments, adherence to guidelines was comparable to cases managed by an onsite-EMS physician. Both teams—one supported by a tele-EMS physician and the other by an onsite-EMS physician—achieved successful blood pressure reductions. In the patient group treated via tele-EMS system, there were fewer cases where a blood pressure drop exceeded the recommended threshold^29^. This finding aligns with the results of our study: only one case reported systolic blood pressure decrease below target values. As this study does not include comparison groups with an onsite-EMS physician, no conclusions can be drawn in this regard. However, the results are consistent with existing literature and support the hypothesis that emergencies in the diagnostic group of hypertensive emergencies can be managed in adherence to guidelines using telemedicine. This conclusion is further supported by another study, which demonstrated that telemedicine-assisted interventions in potentially life-threatening patient conditions led to significant improvements in vital parameters^14^. Based on these results and in consideration of the current body of literature, it can be concluded that the use of the tele-EMS physician system facilitates high-quality therapy within this diagnostic spectrum.

When analyzing the primary respiratory diagnostic group, respiratory infections / pneumonia and obstructive lung diseases emerged as the most frequent diagnoses among the study group. Respiratory infections are highly variable in presentation and thus require diverse treatment approaches^4^. Current studies indicate that COVID-19 infections continue to play an important role in prehospital care, alongside pneumonia and other respiratory tract infections^21,39^. Since these infections require distinct management strategies, and no anti-infective medication were carried in the local EMS, our study investigated oxygen administration. Measured oxygen saturation was significantly increased over the course of the consultation. Obstructive lung diseases were identified as the second most common diagnosis among primary respiratory conditions. For these diagnoses, the administration of ipratropium bromide, salbutamol, prednisone, and oxygen was analyzed. Significant administration rates for the respective medications were observed (*p* < 0.001), representing guideline-adherent treatment. Other studies have shown that such management, in cases involving onsite-EMS physicians or in an in-hospital setting, leads to improved patient outcomes and adheres to current guidelines^40,41^. As our findings are consistent with these results, it can be concluded that treatment of chronic obstructive lung disease within the tele-EMS physician system also ensures a high quality of care. For other diagnostic groups (trauma, primary neurological / psychiatric and other conditions), improvements in vital parameters toward target ranges were also observed. It is noteworthy, that respiratory rate was recorded relatively more frequently in trauma patients compared to the other diagnostic groups. Overall, respiratory rate was recorded less frequently across all diagnostic groups compared to other vital parameters. This vital sign is rarely measured in prehospital EMS care, primarily because only a few monitoring devices are capable of capturing it automatically^42,43^. As a result, there is a gap in the assessment and management of respiratory emergencies. Moving forward, the routine measurement of respiratory rate should become a standard practice during emergency operations. We assume that respiratory rate was recorded more frequently in trauma patients compared to other diagnostic groups, as its assessment is integrated into the structured ABCDE approach commonly used in trauma care.

### Incidents with additional alarm of onsite-EMS physician

An onsite-EMS physician was requested for a total of 14 cases. This corresponds to 0.63% (14/2234) of all cases considered in this study and thus represents a small proportion. These patients showed a higher frequency of critical NACA scores (> / = NACA 4) compared to the overall population examined. Accordingly, the patients in this subgroup can be classified as potentially life-threatening (NACA 4) and in acute risk of death (NACA 5)^14^. All data from these missions demonstrate clear indications for requesting an onsite-EMS physician. For example, securing the airway through endotracheal intubation was necessary in two cases. In Aachen, Germany, an on-site EMS physician is required for this procedure due to their specific expertise^25^. Additionally, the expertise of the on-site EMS physician was required in situations where the paramedics may have lacked full confidence. Furthermore, the tele-EMS physician system is not intended to substitute for an onsite-EMS physician in life-threatening emergencies. However, studies demonstrated that many potentially life-threatening emergencies could be managed by the tele-EMS physician, provided they did not require invasive interventions that would necessitate an onsite-EMS physician^8,14,16^. Nonetheless, the study also highlights that conditions such as myocardial infarctions, cardiac pulmonary edema, and malignant arrhythmias require the presence of an onsite-EMS physician^14^. This aligns with our findings. It was demonstrated that vital parameters remained stable or improved, and the tele-EMS physician system bridged the time until the arrival of the onsite-EMS physician, thereby reducing the physician-led therapy-free interval.

### Limitations

Since this monocentric study examines the tele-EMS system in one location, the results are not generalizable and may not be directly applicable to other regions with different conditions. Further research is needed to investigate the impact of the technical, spatial, and structural conditions of the tele-EMS physician center. For example, whether simultaneous clinical activities (e.g. in an emergency department) might impair the quality and adherence to guideline-based care. Based on the retrospective nature of this study the tele-EMS protocols were not developed exclusively for our analyses. Furthermore, the protocols were completed by the attending tele-EMS physicians directly during and after missions. This may introduce both reporting and recall bias. No arterial blood gas analyses were performed during the consultations. This could have provided further insights for assessing the patient’s condition. Another limitation is the preselection performed by the emergency dispatch center, where decisions are made regarding which resources are dispatched to the scene. In our study, we analyzed the protocols of patients managed via telemedicine. However, the protocols of patients with respiratory emergencies who were primarily treated by an on-scene emergency physician or exclusively by an ambulance were not included. This presents notable potential for future research.

## Conclusion

Overall, patients with respiratory problems included in the study were successfully treated. The application of the tele-EMS physician system demonstrated significant changes in vital parameters, adjusting them into the desired target range. Despite the various diagnoses that can lead to respiratory symptoms, the tele-EMS physicians were able to provide adequate treatment together with on-site paramedics. Guideline-compliant therapies were prescribed and implemented, resulting in enhanced patient outcomes. An analysis of individual diagnoses allowed for categorization into different groups of respiratory issues. These analyses demonstrated that stabilization of vital parameters could also be achieved within these specific groups. Highlighting the individual clinical pictures further substantiated improved outcomes and adherence to guideline-based therapy.

In the future, the individual diagnoses identified here should be further investigated, for example, through an in-hospital follow-up of patients, which could help identify risk constellations.

## Data Availability

The datasets analyzed in the current study are not publicly available because they are municipal property and cannot be published online under open-access agreements. However, these datasets are available upon reasonable request and with permission from municipal authorities. Correspondence and requests for materials should be addressed to A.B..

## Abbreviations

ACS: Acute coronary syndrome
CI: Confidence interval
EMS: Emergency medical service
NACA: National Advisory Committee for Aeronautics
SD: Standard deviation
SpO_2_: Peripheral oxygen saturation
tele-EMS: Telemedicine emergency medical service
